# Identification of HMCES as the Core Genetic Determinant Underlying the *xhs1* Radiosensitivity Locus in LEA/LEC Rats

**DOI:** 10.3390/ijms27031278

**Published:** 2026-01-27

**Authors:** Eisuke Hishida, Masaki Watanabe, Takeru Sasaki, Tatsuya Ashida, Keisuke Shimada, Tadashi Okamura, Takashi Agui, Nobuya Sasaki

**Affiliations:** 1 Laboratory of Laboratory Animal Science and Medicine, School of Veterinary Medicine, Kitasato University, Towada 034-8628, Aomori, Japan; 2 Laboratory of Experimental Animal Science, School of Veterinary Medicine, Rakuno Gakuen University, Ebetsu 069-8501, Hokkaido, Japan; 3 Department of Laboratory Animal Medicine, National Institute of Global Health and Medicine—Japan Institute for Health and Security (JIHS), Shinjuku 162-8655, Tokyo, Japan; 4 Laboratory of Laboratory Animal Science and Medicine, Department of Applied Veterinary Sciences, Graduate School of Veterinary Medicine, Sapporo 060-0819, Hokkaido, Japan

**Keywords:** HMCES, radiosensitivity, DNA double-strand break repair, end-joining repair, *xhs1* locus

## Abstract

Genomic instability caused by defective DNA double-strand break (DSB) repair is a key determinant of cellular radiosensitivity. The Long–Evans cinnamon (LEC) rat is a rare naturally occurring model with marked radiosensitivity, and a major quantitative trait locus, X-ray hypersensitivity 1 (*xhs1*), has been mapped to rat chromosome 4; however, the causal mechanism has remained unclear. Here, we investigated the cellular and molecular basis of *xhs1*-associated radiosensitivity using LEA and LEC rat-derived cells and human cultured cells. Exploratory RNA-seq of pre-hepatitic liver tissue identified a sequence variant within the *Hmces* transcript in LEC rats. Consistently, HMCES protein levels were markedly reduced in multiple tissues and liver-derived cell lines from LEC rats. Functional analyses showed that reduced HMCES activity prolonged γH2AX signaling after X-ray irradiation, indicating delayed DSB resolution. Clonogenic survival assays demonstrated increased radiosensitivity in HMCES-deficient cells, which was partially rescued by restoring HMCES expression in stable LEA/LEC lines. Moreover, pimEJ5GFP reporter assays revealed significantly decreased end-joining repair activity in HMCES-knockout human cells. Together, these results establish HMCES as a critical mediator of DSB repair and cellular radioresistance, identify HMCES dysfunction as a core genetic determinant underlying *xhs1*-associated radiosensitivity, and provide mechanistic insight into radiation response architecture in a naturally occurring radiosensitive model.

## 1. Introduction

Genomic DNA is constantly exposed to a wide variety of damaging agents, including ultraviolet radiation, ionizing radiation, and reactive oxygen species generated during normal cellular metabolism. If such DNA damage is not properly repaired, it can lead to cell death, genomic instability, tumorigenesis, and ultimately organismal lethality. To prevent these outcomes, organisms have evolved highly conserved DNA damage response and repair systems that rapidly detect and resolve DNA lesions [1,2]. Among these lesions, DNA double-strand breaks (DSBs) are considered the most cytotoxic, and the capacity to repair DSBs is a critical determinant of radiosensitivity [3,4].

Radiosensitivity is defined as differential cellular, tissue, or organismal responses to the same dose of ionizing radiation. Elucidation of its molecular basis is of fundamental importance for optimizing radiotherapy and improving radiation protection strategies [1,2]. Previous studies have demonstrated that defects in DSB repair pathways, particularly non-homologous end joining (NHEJ) and homologous recombination (HR), are closely associated with increased radiosensitivity [1,2,3,4,5].

The Long–Evans cinnamon (LEC) rat is well known as a model of spontaneous hepatitis and hepatocellular carcinoma, but it also represents a rare naturally occurring model of marked radiosensitivity compared with other rat strains [6,7,8]. Both LEC rats and the closely related Long–Evans agouti (LEA) rats exhibit severe damage to hematopoietic and intestinal epithelial tissues even after relatively low doses of X-ray irradiation, resulting in a significantly reduced lethal dose threshold [8,9]. These characteristics make LEC/LEA rats a valuable experimental system for investigating genetically determined abnormalities in radiation responses.

Quantitative trait locus analyses have suggested that a major genetic locus that is responsible for the radiosensitive phenotype in LEC/LEA rats, designated X-ray hypersensitivity 1 (*xhs1*), is located on rat chromosome 4 [7,9]. Tsujii et al. narrowed this *xhs1* locus to an approximately 1.2 Mbp region by generating congenic rats and evaluating their radiosensitivity phenotypes [10]. This genomic interval contains multiple genes, indicating that functional analyses of genes within this region are essential for understanding the molecular basis of radiosensitivity.

Previous studies have further reported abnormalities in DNA damage responses in cells derived from LEC and LEA rats, particularly in processes following DSB induction [7,9]. However, how genetic alterations within the *xhs1* region influence post-irradiation DNA damage signaling, cellular survival, and end-joining repair activity has not been systematically investigated at the cellular level.

Among the major DSB repair pathways, end joining plays a predominant role in the repair of ionizing radiation-induced DSBs, particularly in non-replicating or slowly proliferating cells, and operates throughout the cell cycle [4,5]. In contrast to homologous recombination, which is restricted to S and G2 phases and requires an intact sister chromatid as a repair template, end-joining pathways provide a rapid and broadly available mechanism for restoring DNA integrity following irradiation. Therefore, alterations in end-joining capacity are expected to have a direct and substantial impact on cellular radiosensitivity. Based on this biological context, and given prior evidence of post-DSB response abnormalities in LEC- and LEA-derived cells, the present study focused on end-joining repair activity as a functionally relevant readout to interrogate genetically determined differences in radiation responses associated with the *xhs1* locus.

In addition, in studies aimed at dissecting genetically determined differences in cellular radiosensitivity, not only the total radiation dose but also the dose rate is an important experimental parameter. Dose rate influences the temporal relationship between DNA damage induction and repair, thereby affecting the sensitivity with which defects in DNA damage signaling and repair progression can be detected. Extremely high dose rates may overwhelm cellular repair systems and induce rapid cytotoxicity, whereas excessively low dose rates can permit substantial repair during irradiation, potentially masking intrinsic differences in post-irradiation responses.

In the present study, LEA- and LEC-derived cells, together with human cultured cells, were used to comprehensively analyze DNA damage responses after X-ray irradiation, cellular survival, and end-joining repair activity. Through these analyses, this study aimed to deepen the understanding of the cellular and molecular mechanisms underlying radiosensitivity associated with the *xhs1* locus in a naturally occurring radiosensitive model.

## 2. Results

### 2.1. RNA-Seq Analysis Identified an Hmces Variant and Expression Changes in Candidate Genes Within the xhs1 Locus

**Figure 1:**
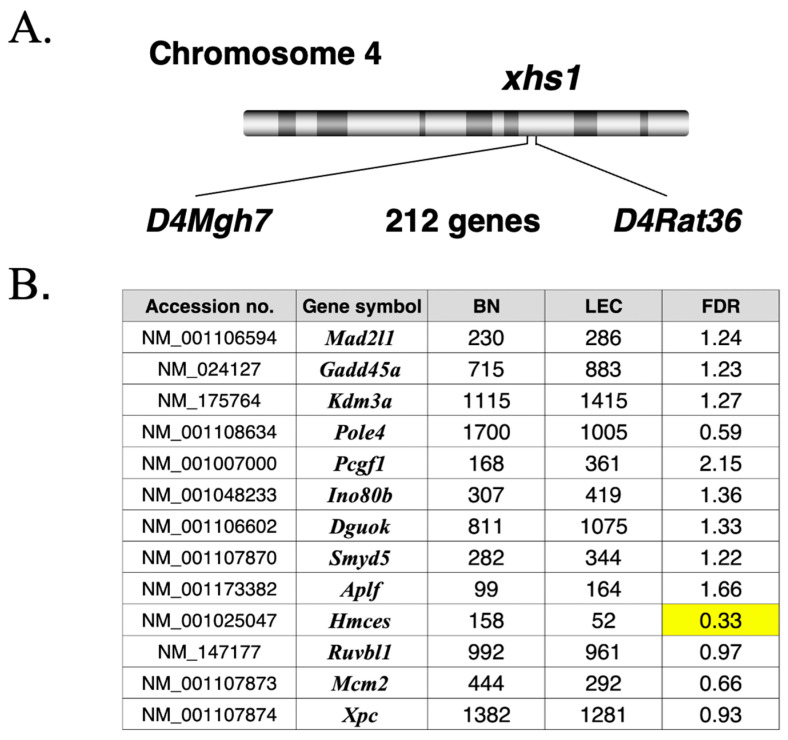
RNA-seq-based identification of an *Hmces* variant and candidate gene expression changes within the *xhs1* locus. (**A**) Schematic representation of the *xhs1* congenic interval on rat chromosome 4 defined by the microsatellite markers *D4Mgh7* and *D4Rat36*, encompassing 212 annotated genes. (**B**) Exploratory RNA-seq analysis of liver samples from 4-week-old male BN and LEC rats (*n* = 1 per strain; LEC sampled prior to hepatitis onset). Normalized read counts in BN and LEC rats, fold change (LEC/BN), and false discovery rate (FDR) values are shown for representative DNA repair- and genome maintenance-related genes located within the interval, including *Hmces*. The FDR value for *Hmces* is highlighted in yellow.

To explore candidate genes underlying the radiosensitive phenotype associated with the *xhs1* locus, we performed an exploratory RNA-seq analysis using liver tissue from 4-week-old male BN and LEC rats (*n* = 1 per strain), with the LEC sample collected prior to hepatitis onset. The *xhs1* congenic interval on rat chromosome 4 was defined by the microsatellite markers *D4Mgh7* and *D4Rat36* and contains 212 annotated genes (Figure 1A). Sequence reads were mapped to the rat reference genome, candidate variants were extracted across the *Hmces* transcript region, and differential expression analysis was performed using DESeq2 with Benjamini–Hochberg adjustment [11,12,13]. Differential expression results for all 212 genes located within the interval, including normalized read counts, fold change (LEC/BN), and false discovery rate (FDR), are provided in Supplementary Table S1.

RNA-seq analysis was performed to identify candidate genes within the mapped interval. In parallel, we curated candidate genes related to DNA repair and genome maintenance within the interval based on public functional annotations, including Gene Ontology terms associated with DNA repair and DNA damage response (e.g., GO:0006281 and related descendant terms) (Figure 1B). Among these curated candidates, *Hmces* showed a marked reduction in expression in LEC relative to BN (normalized counts 158 vs. 52; fold change 0.33), whereas several other candidates exhibited modest increases or decreases (Figure 1B). Because the sample size was limited (*n* = 1 per strain), this RNA-seq dataset was used only for candidate prioritization (hypothesis generation), not for quantitative inference. Therefore, FDR values are reported descriptively and were not used to claim statistical significance. Nonetheless, the convergence of an *Hmces* transcript variant and reduced *Hmces* expression provided a molecular rationale for subsequent functional analyses focusing on HMCES and radiation-related cellular phenotypes.

### 2.2. Identification of an Hmces Nonsense Variant and Reduced HMCES Protein Expression in LEC Rats

**Figure 2:**
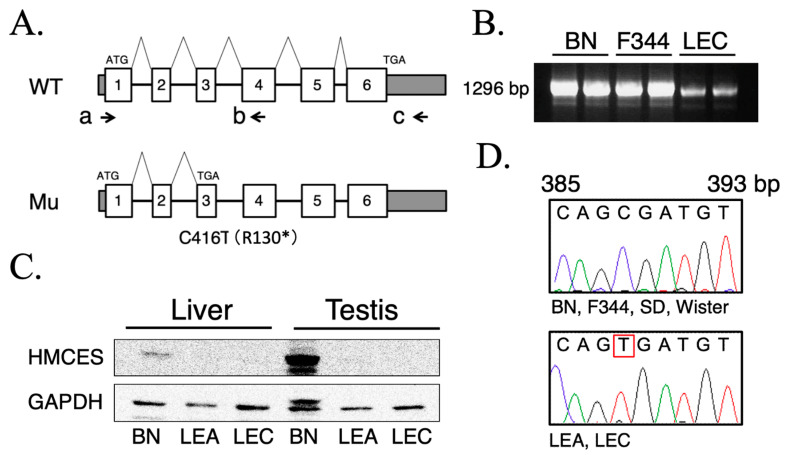
Identification and validation of an *Hmces* variant in LEC rats. (**A**) Schematic representation of the *Hmces* gene structure and primer design used for RT-PCR and sequencing analyses. The wild-type (WT) allele consists of six exons, whereas the mutant (Mu) allele identified in LEC rats carries a C416T nucleotide substitution, resulting in a premature stop codon (R130*). Arrows indicate the positions of primers used for RT-PCR amplification and sequencing: F1 (a), mid R2 (b), and R1 (c). (**B**) RT-PCR analysis of *Hmces* transcripts amplified from liver cDNA of BN, F344, and LEC rats using primers F1 and R1. A single band of the expected size (1296 bp) was detected in all strains, indicating comparable transcript length and successful amplification. These analyses were performed using at least three independent biological replicates for each experimental condition, with each sample measured once (no technical replicates were performed). (**C**) Western blot analysis of HMCES protein expression in liver and testis tissues from BN, LEA, and LEC rats. While HMCES expression was detected in the liver and testis of BN rats, the protein was markedly reduced in these tissues in both LEA and LEC strains. GAPDH was used as a loading control. These analyses were performed using at least three independent biological replicates for each experimental condition, with each sample measured once (no technical replicates were performed). (**D**) Representative Sanger sequencing chromatograms of *Hmces* cDNA. Upper panel shows the WT sequence detected in BN, F344, SD, and Wistar rats. Lower panel shows a C→T transition (red box) identified in LEA and LEC rats, corresponding to the C416T substitution that introduces a premature stop codon. Sequences were aligned and compared with the rat *Hmces* reference sequence (NM_001025047.2).

To determine whether the candidate alteration detected by RNA-seq reflected a bona fide genetic variant and affected HMCES expression, we next performed molecular and biochemical analyses of *Hmces* in multiple rat strains and tissues. To assess transcript integrity and abundance, RT-PCR was performed using liver cDNA from BN, F344, and LEC rats with primers F1 and R1 spanning the *Hmces* coding region (Figure 2A). A single amplification product of the expected size (1296 bp) was detected in all strains examined (Figure 2B), indicating that the overall transcript length of *Hmces* was preserved and that gross aberrant splicing was not evident. Notably, the RT-PCR band appeared less intense in LEC than in BN or F344 under the same amplification conditions, suggesting reduced steady-state *Hmces* mRNA abundance in LEC liver. This qualitative reduction was consistent with the RNA-seq result showing decreased *Hmces* expression in LEC relative to BN (Figure 1B). Direct Sanger sequencing of the RT-PCR products revealed a C→T nucleotide substitution at position 416 (C416T) in *Hmces* cDNA specifically in LEA and LEC rats, both of which exhibit pronounced radiosensitivity (Figure 2D). In contrast, the wild-type sequence was consistently detected in BN, F344, SD, and Wistar rats, which are relatively radioresistant under comparable conditions. This clear strain-dependent segregation indicates that the *Hmces* C416T mutation co-segregates with the radiosensitive phenotype across rat strains. The C416T substitution introduces a premature stop codon (R130*) within the HMCES coding sequence (Figure 2A). We next examined whether this nonsense variant was associated with altered HMCES protein expression. Immunoblot analysis showed that HMCES protein was detectable in the liver and testis of BN rats, but was markedly reduced in both tissues in LEA and LEC rats (Figure 2C). In contrast, although HMCES was robustly expressed in the testis of radioresistant strains, HMCES protein was markedly reduced or undetectable in the testis of LEA and LEC rats, indicating tissue-dependent expression patterns but a strain-dependent loss of HMCES expression. Together, these results demonstrate that radiosensitive rat strains harbor a nonsense variant in *Hmces* that is associated with reduced *Hmces* transcript abundance and markedly diminished HMCES protein expression in liver tissue and liver-derived cell lines.

### 2.3. HMCES Restoration Partially Rescues Cellular Radiosensitivity in LEA and LEC Cells

**Figure 3:**
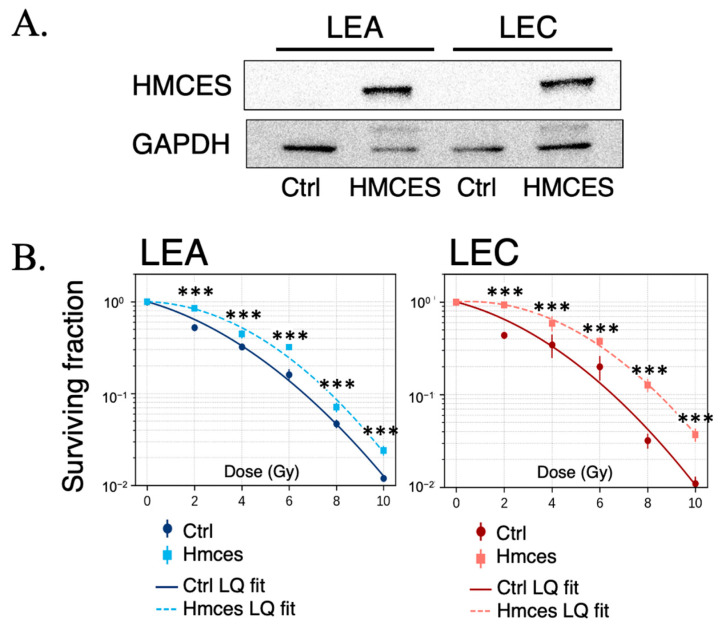
Clonogenic survival of LEA and LEC cells fitted with the linear–quadratic (LQ) model. (**A**) Validation of HMCES expression in stable HMCES-expressing LEA/LEC cell lines and control (Ctrl) vector by immunoblot; GAPDH, loading control. These analyses were performed using at least three independent biological replicates for each experimental condition, with each sample measured once (no technical replicates). (**B**) Surviving fraction (SF) after X-ray irradiation plotted against dose (Gy). Data are mean ± SD (*n* = 6 independent biological replicates). Curves show LQ model fits: SF(D) = exp(−αD − βD^2^). Statistical comparisons between Ctrl and HMCES groups at each dose were performed using Welch’s two-sided *t*-test with Holm’s adjustment. Significance: *** *p* < 0.001. Abbreviations: LQ, linear–quadratic; Ctrl, control; HMCES, rat HMCES protein.

To directly assess the functional contribution of HMCES to cellular radiation responses, we established stable HMCES-expressing cell lines derived from LEA and LEC rats. Western blot analysis confirmed robust expression of HMCES protein in both LEA-HMCES and LEC-HMCES cells compared with their respective control counterparts, while GAPDH levels were comparable across samples, indicating equivalent protein loading (Figure 3A). We next evaluated the impact of HMCES expression on cellular radiosensitivity using clonogenic survival assays following X-ray irradiation. In LEA-derived cells, stable expression of HMCES tended to increase the surviving fraction across a range of radiation doses compared with control cells (Figure 3B, left). Similarly, in LEC-derived cells, which exhibited pronounced radiosensitivity in the control condition, re-expression of HMCES significantly improved clonogenic survival at all tested doses (Figure 3B, right).

Survival curves were fitted using the linear–quadratic (LQ) model, and statistically significant differences between control and HMCES-expressing cells were observed at multiple dose points (in LEC cells; *** *p* < 0.001). Collectively, these results indicate that restoration of HMCES expression increases resistance to ionizing radiation, with a more evident effect in LEC cells.

### 2.4. Altered DNA Damage Response Kinetics After Irradiation in Cells with Reduced HMCES Function

**Figure 4:**
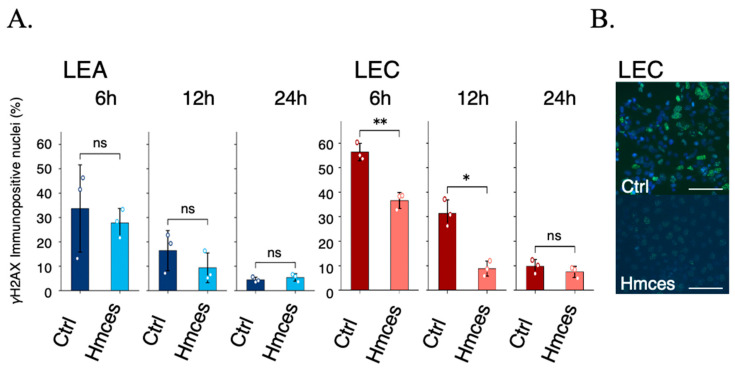
γH2AX-based DNA damage response after X-ray irradiation. (**A**) LEA and LEC cells carrying a control vector (Ctrl) or stably expressing HMCES (Hmces) were irradiated with X-rays (2 Gy) and analyzed at 6, 12, and 24 h. γH2AX immunofluorescence was quantified as the percentage of γH2AX-positive nuclei among total DAPI-positive nuclei. Data are shown as mean ± SD from three independent biological experiments; open circles indicate individual replicates. Because different cell populations were fixed at each time point, comparisons were performed independently at each time point rather than as repeated measures. (**B**) Representative γH2AX (green) and DAPI (blue) images of LEC cells at 6 h after irradiation. Scale bars, 100 μm. Statistical comparisons between Ctrl and Hmces were performed at each time point using Welch’s two-sided *t*-test. * *p* < 0.05, ** *p* < 0.01; ns, not significant.

To investigate whether HMCES influences the early DNA damage response after ionizing radiation, we analyzed γH2AX formation following X-ray irradiation. γH2AX was used as a sensitive marker of DNA double-strand breaks (DSBs) and their repair kinetics [14,15]. LEA and LEC cells carrying either the control vector (Ctrl) or stably expressing HMCES (*Hmces*) were exposed to 2 Gy X-ray irradiation and analyzed at 6, 12, and 24 h after irradiation (Figure 4). γH2AX immunofluorescence was quantified as the percentage of γH2AX-positive nuclei relative to total DAPI-positive nuclei, using ImageJ (1.54p) under fixed imaging conditions and a predefined intensity threshold (maximum nuclear intensity ≥ 1800) empirically determined from non-irradiated controls [16]. Data represent mean ± SD from three independent biological experiments, each performed with at least two wells per condition and time point; individual biological replicates are shown as open circles. Quantitative analysis revealed clear strain- and HMCES-dependent differences in γH2AX kinetics (Figure 4A). In LEA cells, stable expression of HMCES did not significantly alter the fraction of γH2AX-positive nuclei at any time point (6–24 h; ns), indicating only a modest effect under this condition. In contrast, LEC Ctrl cells exhibited a high proportion of γH2AX-positive nuclei at early time points, and HMCES expression significantly reduced γH2AX positivity at 6 h (*p* < 0.01) and 12 h (*p* < 0.05), whereas the difference was no longer significant at 24 h (ns) (Welch’s two-sided *t*-test performed independently at each time point). Representative γH2AX images at 6 h in LEC cells (Ctrl vs. *Hmces*) illustrate the reduced γH2AX signal intensity/positivity upon HMCES expression (Figure 4B). Together, these findings indicate that reduced HMCES function is associated with prolonged or elevated DNA damage signaling after irradiation, and that restoring HMCES in LEC cells accelerates early γH2AX resolution, consistent with impaired processing and/or resolution of radiation-induced DSBs in the HMCES-deficient state.

### 2.5. HMCES Modulates Cellular Radiosensitivity and End-Joining Repair Activity

**Figure 5:**
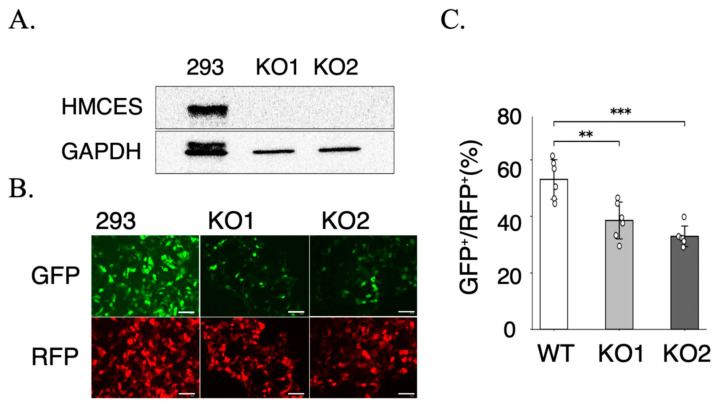
Reduced end-joining activity in HMCES-knockout 293 cells assessed with the pimEJ5GFP reporter. (**A**) Immunoblot of HMCES in WT and two independent knockout clones (KO1, KO2); GAPDH, loading control. These analyses were performed using at least three independent biological replicates for each experimental condition, with each sample measured once (no technical replicates). (**B**) Representative fluorescence images 48 h after transfection (GFP, end-joining–dependent signal; RFP, transfection control). Scale bars, 100 μm. (**C**) End-joining activity quantified as GFP^+^/RFP^+^ (%). Data are mean ± SD (*n* = 6). One-way ANOVA with Holm-adjusted planned comparisons versus WT. ** *p* < 0.01; *** *p* < 0.001.

To assess the functional consequences of altered DNA damage responses, we next examined cellular survival after irradiation using clonogenic assays. LEA and LEC cells displayed distinct radiosensitivity profiles, with LEC cells showing significantly reduced surviving fractions following X-ray irradiation (Figure 3B). Notably, stable expression of HMCES in LEC cells partially restored clonogenic survival, indicating that HMCES contributes to radioresistance at the cellular level. To further explore the underlying DNA repair mechanism, we evaluated DSB end-joining activity using the pimEJ5GFP reporter system, in which GFP expression is dependent on end-joining, while RFP serves as a transfection control [4,5]. To provide complementary cross-species support, we used 293 cells to specifically quantify DSB end-joining capacity using this reporter; irradiation survival/radiosensitivity analyses were not performed in this system. HMCES knockout in 293 cells was verified by immunoblotting in two independent clones (KO1 and KO2), confirming marked depletion of HMCES protein relative to the parental wild-type (WT) cells (Figure 5A). Forty-eight hours after reporter transfection, representative fluorescence images showed reduced GFP signal in KO cells compared with WT, whereas RFP expression was preserved (Figure 5B). End-joining activity was quantified as the percentage of GFP^+^/RFP^+^ cells, demonstrating a significant reduction in both KO1 and KO2 compared with WT (mean ± SD, *n* = 6) (Figure 5C). Statistical analysis using one-way ANOVA followed by planned comparisons versus WT with Holm’s multiple-comparison adjustment confirmed decreased end-joining efficiency in KO cells (*p* < 0.01 for KO1 vs. WT; *p* < 0.001 for KO2 vs. WT). Together, the clonogenic survival assays in LEA/LEC cells and the pimEJ5GFP assays in 293 cells indicate that HMCES promotes efficient DSB end-joining repair, consistent with enhanced cellular survival following irradiation.The concordance between increased radiosensitivity and reduced end-joining activity supports a model in which HMCES contributes to the maintenance of genomic integrity after DSB induction. The partial, but not complete, rescue of clonogenic survival is consistent with HMCES being a major, but not exclusive, determinant of radiosensitivity, potentially alongside additional modifier loci such as *xhs2.* Although the pimEJ5GFP reporter primarily reflects classical non-homologous end joining (c-NHEJ), the reduced GFP^+^/RFP^+^ fraction in HMCES-deficient cells suggests that HMCES contributes to end-joining capacity, either directly or indirectly, by facilitating efficient processing of DSB ends [4,5].

## 3. Discussion

In this study, we investigated the molecular basis of radiosensitivity associated with the *xhs1* locus using naturally occurring radiosensitive LEA and LEC rat strains and their derived cell lines. By stepwise integration of exploratory RNA-seq analysis (Figure 1), protein expression profiling (Figure 2), DNA damage response analysis (Figure 4), and DNA repair functional assays (Figure 5), we demonstrate that HMCES functions as a core, albeit not sole, genetic determinant of cellular responses to ionizing radiation and radioresistance.

Radiosensitivity in LEC and LEA rats has been shown by previous genetic studies to be strongly linked to the *xhs1* locus on rat chromosome 4 [7,9]. This locus exhibits an exceptionally high LOD score (LOD = 115.4), indicating that it harbors major genetic determinants of radiosensitivity [10].

The *Hmces* nonsense variant and reduced expression identified in the present study (Figure 1) can be positioned as molecular abnormalities originating from this *xhs1* region, supporting the interpretation that HMCES represents a central component underlying radiosensitivity in these strains.

Although the RNA-seq analysis performed in this study was exploratory in nature and limited by sample size, and therefore not intended for quantitative transcriptomic inference, its application to pre-hepatitic LEC rat liver provided a rational framework for prioritizing candidate genes within the *xhs1* interval (Figure 1) [11]. Importantly, the consistent reduction in HMCES protein levels across multiple tissues and liver-derived cell lines from LEC rats (Figure 2) indicates that HMCES dysregulation reflects a systemic, strain-dependent characteristic rather than a secondary consequence of tissue pathology or inflammation.

HMCES has previously been characterized primarily as a DNA damage tolerance factor involved in the response to abasic (AP) sites and replication-associated lesions [17,18]. In particular, HMCES forms covalent DNA–protein crosslinks at AP sites, thereby protecting stalled replication forks from collapse and preventing secondary double-strand break (DSB) formation [17,18]. On this basis, HMCES has been positioned mainly as a replication stress-associated genome maintenance factor.

In contrast, our findings indicate that the function of HMCES extends beyond replication-associated damage responses and contributes to cellular responses to DSBs directly induced by ionizing radiation. LEC cells exhibited prolonged γH2AX signaling following X-ray irradiation (Figure 4), and stable expression of HMCES partially alleviated this persistence, suggesting that HMCES contributes to post-irradiation damage processing and repair progression.

Importantly, the effect of HMCES was observed in the persistence rather than the initial induction of γH2AX signals, arguing against a primary role in DSB sensing or upstream ATM-dependent signaling per se. As γH2AX is a well-established marker reflecting DSB formation and repair kinetics [14,15,19], these results are consistent with impaired processing and/or resolution of radiation-induced DSBs in HMCES-deficient cells.

Consistent with these observations, clonogenic survival assays demonstrated that restoration of HMCES expression partially rescued radiosensitivity in LEC cells (Figure 3B) [20,21]. Furthermore, in human 293 HMCES-knockout cells used as a complementary cross-species model, functional analysis using the pimEJ5GFP reporter system revealed a significant reduction in end-joining repair activity in HMCES-deficient cells (Figure 5B,C) [22]; irradiation survival/radiosensitivity was not directly addressed in this system. Because end joining represents a major pathway for the repair of radiation-induced DSBs [4,5], these results together with the clonogenic survival rescue observed in LEC cells support a functional role for HMCES in maintaining DSB repair capacity and cellular radioresistance. Although the pimEJ5GFP reporter primarily reflects classical non-homologous end joining (c-NHEJ), this assay does not exclude additional contributions of HMCES to alternative end-joining pathways or repair pathway choice, which were not directly addressed in the present study [23].

Taken together, these findings suggest that HMCES is not a factor specialized for a single class of DNA lesions but rather an integrative component positioned at the interface between damage processing and repair pathway control. In this context, HMCES may contribute to genome stability by facilitating the orderly progression of repair reactions across distinct damage contexts, including both replication-associated lesions and radiation-induced DSBs.

In interpreting these findings, it is also important to consider the strain history of LEA and LEC rats. These strains were established from multiple closed-colony rats transferred in 1975 from the Kobe University School of Medicine to Hokkaido University, followed by strain separation and fixation [24]. In light of this history, the *Hmces* nonsense variant identified in this study (Figure 1) is unlikely to have arisen de novo in the LEC strain, but rather may represent a pre-existing background mutation that became fixed during strain establishment. This strain-historical context supports the interpretation that HMCES dysfunction represents a foundational genetic component of radiosensitivity associated with the *xhs1* locus, rather than an incidental or strain-specific alteration. A plausible mechanistic explanation for the reduced *Hmces* mRNA levels is nonsense-mediated mRNA decay (NMD), a translation-dependent surveillance pathway that selectively degrades transcripts harboring premature termination codons (PTCs) [25,26,27]. Thus, the combination of a nonsense variant and reduced *Hmces* transcript abundance strongly suggests that *Hmces* mRNA is destabilized via NMD in LEC rats. Despite their shared origin, LEA and LEC rats exhibit clear differences in the severity of radiosensitivity [9]. While LEA rats display increased radiosensitivity, its magnitude is substantially milder than that observed in LEC rats, indicating that *xhs1* alone cannot fully account for the phenotypic difference. In this regard, a second radiosensitivity-related locus, *xhs2*, has been identified on rat chromosome 1 specifically in the LEC strain, with a reported LOD score of 14.8 [9]. Although the causative gene at the *xhs2* locus remains unknown, the marked difference in LOD scores between *xhs1* and *xhs2* supports a hierarchical model in which *xhs1* (*Hmces*) functions as the core determinant of radiosensitivity, while *xhs2* and potentially other loci act as secondary modifiers that enhance or exacerbate the phenotype. Thus, LEA rats may represent a baseline *xhs1*-dependent radiosensitive state, whereas LEC rats exhibit an exacerbated phenotype due to additional modifier loci such as *xhs2*. Accordingly, the relatively mild radiosensitivity observed in LEA rats may reflect the presence of a shared *xhs1*-derived baseline susceptibility in the absence of additional modifying effects from *xhs2* or other loci. Radiosensitivity in LEA and LEC rats is therefore best understood as a multifactorial trait shaped by hierarchical and combinatorial genetic effects.

Several limitations of the present study should be acknowledged. First, the RNA-seq analysis was exploratory in nature and limited by sample size, and thus was not intended to provide comprehensive transcriptomic inference. Moreover, direct interactions between HMCES and core end-joining factors have not yet been defined. Future studies employing molecular interaction analyses, pathway-specific repair assays, and genetically engineered animal models will be required to clarify the mechanistic role of HMCES in DSB repair and to establish its contribution to organismal radiosensitivity in vivo [28].

In conclusion, this study demonstrates that HMCES is a core genetic determinant underlying radiosensitivity associated with the *xhs1* locus, contributing to DNA double-strand break repair capacity, cellular radioresistance, and maintenance of genomic integrity. While the RNA-seq analysis employed in this study was exploratory and limited by sample size, integration with complementary molecular and functional analyses enabled robust identification of HMCES as a key component of radiosensitivity.

## 4. Materials and Methods

### 4.1. Ethical Statement

All animal experiments were conducted in accordance with the regulations for the care and use of laboratory animals at Kitasato University and Hokkaido University.

The animal study protocol was reviewed and approved by the Institutional Animal Care and Use Committee of Kitasato University and authorized by the President of Kitasato University (approval no. 25-072) and was additionally reviewed by the Institutional Animal Care and Use Committee of Hokkaido University and authorized by the President of Hokkaido University (approval no. 08-0384).

### 4.2. Animals and Tissue Collection

BN rats were purchased from Charles River Laboratories Japan (Yokohama, Japan). F344, SD, and Wistar rats used as control strains were purchased from Japan CLEA Co., Ltd. (Tokyo, Japan). LEC and LEA rats were provided by the Department of Biological Sciences, Faculty of Science, Hokkaido University. Both male and female animals at 4 weeks of age were used for all strains. Animals were euthanized under inhalation anesthesia with isoflurane (DS Pharma, Osaka, Japan). The brain, heart, lungs, spleen, liver, kidneys, skeletal muscle, small intestine, ovaries, and testes were collected and used for RNA or protein extraction.

### 4.3. RNA Sequencing Analysis

To identify *Hmces* variants and to characterize expression changes in candidate genes, total RNA was extracted from the livers of BN and LEC rats and subjected to RNA-seq analysis. Male rats at 4 weeks of age were used, with *n* = 1 per strain (BN, *n* = 1; LEC, *n* = 1). All LEC samples were obtained prior to the onset of hepatitis and from non-irradiated animals. Total RNA was extracted using NucleoSpin RNA (Macherey-Nagel, Düren, Germany), and library preparation and sequencing were outsourced to a commercial provider (Nippon Genetics Co., Ltd., Tokyo, Japan). Paired-end sequencing (2 × 150 bp) was performed with a target output of approximately 6 Gbp per sample. Raw data were received in FASTQ format, followed by in-house quality assessment and filtering, and then mapped to the rat reference genome [13]. Candidate variants were extracted based on sequence information across the *Hmces* transcript region. Differential expression analysis was performed using DESeq2 for normalization and estimation of expression changes [11], and statistical significance was evaluated using Benjamini–Hochberg-adjusted *p*-values (FDR) to correct for multiple testing [12]. This analysis was positioned as an exploratory analysis aimed at prioritizing candidate genes, and results were interpreted in light of the limited sample size. In the Section 2, fold change (LEC/BN) is reported together with FDR values.

### 4.4. RT-PCR Expression Analysis and Sequence Analysis of Candidate Genes

Total RNA was extracted from liver tissues of BN, F344, and LEC rats using TRIzol Reagent (Life Technologies, Carlsbad, CA, USA) according to the manufacturer’s instructions. One microgram of total RNA was mixed with 5 pg of oligo(dT) primer and DEPC-treated distilled water to a final volume of 13 μL. The mixture was heated at 70 °C for 10 min and then cooled on ice to allow for primer annealing. Reverse transcription was performed by adding 4 μL of 5× RT reaction buffer, 2 μL of 10 mM dNTP mix, and 1 μL of ReverTra Ace reverse transcriptase (Toyobo, Osaka, Japan). The reaction was incubated at 42 °C for 60 min, followed by enzyme inactivation at 99 °C for 5 min to synthesize cDNA. To examine the presence of sequence variations in the *Hmces* gene between BN and LEC rats, cDNA was amplified using primers F1 (a): 5′-CTGGTGGTCTGAGAGGCATTG-3′ and R1 (c): 5′-TAGCAAGGAGCCAGGGATAG-3′. Direct sequencing was performed using these primers as well as an internal primer, mid R2 (b): 5′-GTAGAGACCTCACCAAAGTCA-3′, covering the 5′ region, central region, and 3′ region of the *Hmces* cDNA. Obtained sequences were aligned and compared with the reference rat *Hmces* sequence registered in the NCBI database (NM_001025047.2) to identify nucleotide substitutions.

### 4.5. Cell Lines and Culture Conditions

Immortalized rat liver-derived cell lines (LEA and LEC) and the human fetal kidney-derived cell line 293 (Thermo Fisher Scientific, Waltham, MA, USA) were used in this study. The immortalized LEA/LEC liver cells were provided by Kumamoto University [9]. All cells were cultured in DMEM High Glucose (Nacalai Tesque, Kyoto, Japan) supplemented with 10% fetal bovine serum (FBS) (Invitrogen, Thermo Fisher Scientific) and 1% penicillin/streptomycin (FUJIFILM Wako Pure Chemical, Osaka, Japan) at 37 °C in a humidified incubator with 5% CO_2_. For selection and maintenance of LEA/LEC cells, Geneticin (G418) (Sigma-Aldrich, St. Louis, MO, USA) was used at 500 µg/mL during establishment and 300 µg/mL for maintenance.

### 4.6. Generation of Hmces Knockout Cells (293 HMCES-KO) by CRISPR/Cas9

Mutations were introduced into the *Hmces* locus in 293 cells using the CRISPR/Cas9 system [29]. The all-in-one vector pSpCas9(BB)-2A-Puro (pX459; Addgene plasmid #62988, Watertown, MA, USA) was used, and an sgRNA expression cassette targeting 5′-CACGAGAGCTTGCGCCTACC-3′ was constructed. Insertion of the sgRNA sequence was confirmed by Sanger sequencing. Approximately 1 × 10^6^ 293 WT cells were seeded in a 10-cm dish, and 24 h later the vector (15 µg) was transfected using TransIT-X2 Reagent (Mirus Bio, Madison, WI, USA). After 48 h, the medium was replaced with puromycin-containing medium (FUJIFILM Wako Pure Chemical, Osaka, Japan) for drug selection. After selection, single-cell clones were isolated from surviving cells and expanded in 96-well plates. Genomic DNA was extracted from each clone, and the target region was amplified by PCR using primers F (5′-TGCGTTCGTGTGTGCACGCGCG-3′) and R (5′-GACATTATATGTGTCCTATAATG-3′). The PCR products were subjected to Sanger sequencing using the F primer to identify indel mutations. Clones predicted to harbor frameshift mutations were selected as knockout candidates, and loss of HMCES protein expression was confirmed by Western blotting. The validated clone was used as 293 HMCES-KO.

### 4.7. Generation of Stable HMCES-Expressing Cells

Rat *Hmces* cDNA was inserted into the pEB Multi-neo vector (FUJIFILM Wako Pure Chemical, Osaka, Japan) to generate the expression plasmid pEB Multi-HMCES. pEB Multi-neo is an episomal expression vector harboring EBV-derived oriP/EBNA1, which enables extrachromosomal replication and maintenance in mammalian cells and thus stable expression without genomic integration [30]. This system also enables efficient generation of polyclonal stable cell populations without the need for single-cell cloning. pEB Multi-HMCES was introduced into LEA/LEC cells, and stable cell lines (LEA-HMCES and LEC-HMCES) were established by selection with Geneticin (G418) (Sigma-Aldrich, St. Louis, MO, USA). Stable lines were maintained in medium containing G418 (300 µg/mL).

### 4.8. Western Blotting

Western blotting was performed using (i) 293 WT and 293 HMCES-KO cells, (ii) LEA/LEC cells and their corresponding stable HMCES-expressing lines, and (iii) liver and testes from BN/LEA/LEC rats. Cultured cells were harvested with TrypLE Express (Invitrogen, Thermo Fisher Scientific, Waltham, MA, USA), washed with PBS, and lysed in RIPA Lysis Buffer (ATTO, Tokyo, Japan) supplemented with protease and phosphatase inhibitors (ATTO, Tokyo, Japan). Tissue samples were homogenized in the same lysis buffer using bead beating, followed by sonication (10 s × 3), and centrifuged at 4 °C to collect the supernatant. An equal volume of 2× sample buffer (0.125 M Tris-HCl, 10% 2-mercaptoethanol, 4% SDS, 10% sucrose, 0.004% bromophenol blue) was added to obtain final 1× concentrations (62.5 mM Tris-HCl, 5% 2-mercaptoethanol, 2% SDS, 5% sucrose, 0.002% bromophenol blue). Samples were denatured at 60 °C for 10 min to preserve antigenicity. Proteins were separated by 10% SDS-PAGE (50 V for 30 min, then 100 V for 1.5 h) and transferred to PVDF membranes (Amersham Hybond-P PVDF membrane; Cytiva, Marlborough, MA, USA) under wet transfer conditions (300 mA, up to 120 V, 120 min). Membranes were blocked with Bullet Blocking One for Western Blotting (Nacalai Tesque, Kyoto, Japan) for 15 min. Primary antibody incubation was performed overnight at 4 °C using rabbit anti-HMCES IgG (1:1000; Sigma-Aldrich, St. Louis, MO, USA) diluted in Signal Booster Solution A (Beacle, Kyoto, Japan). After washing with TBST (3 × 5 min), membranes were incubated with HRP-conjugated anti-rabbit IgG (1:10,000; Nichirei Biosciences, Tokyo, Japan) diluted in Signal Booster Solution B (Beacle, Kyoto, Japan) for 1 h at room temperature. After washing with TBST (3 × 5 min), signals were developed using ECL Prime Western Blotting Detection Reagent (Cytiva, Marlborough, MA, USA) and detected with an Omega Lum C imaging system (v2.1.1027.0, Aplegen, Pleasanton, CA, USA). GAPDH was used as a loading control. Rabbit anti-GAPDH IgG (1:10,000; Sigma-Aldrich, St. Louis, MO, USA) and HRP-conjugated anti-rabbit IgG (1:10,000; Nichirei Biosciences, Tokyo, Japan) were used under the same conditions. These analyses were performed using at least three independent biological replicates for each experimental condition. Technical replicates were not performed unless otherwise stated.

### 4.9. DNA Damage Response Assay by γH2AX Immunofluorescence After X-Ray Irradiation

LEA/LEC cells and their stable HMCES-expressing lines were used. Cells were dissociated into single cells with TrypLE Express (Invitrogen, Thermo Fisher Scientific, Waltham, MA, USA) and seeded into µ-Slide 8 Well chambers (ibidi, Gräfelfing, Germany) at approximately 1 × 10^4^ cells per well in 300 µL medium. After 24 h to allow for attachment, cells were exposed to X-ray irradiation. Irradiation was performed using an X-ray irradiator (MX-80Labo, mediXtec, Matsudo, Japan) at room temperature while rotating on a turntable (80 kVp, 1.25 mA, no filter, irradiation distance 180 mm). The dose was set to 2 Gy according to the device display, with an irradiation time of 282 s. Non-irradiated controls (0 Gy) were handled identically except for the irradiation step. The dose rate used in this study (approximately 0.43 Gy/min) was selected because it falls within a commonly adopted range for in vitro radiobiological assays, while avoiding excessive acute cytotoxicity and enabling discrimination of DNA repair phenotypes [20,21]. In this study, the aim was not to reproduce clinical radiotherapy conditions but rather to compare intrinsic radiosensitivity between genetically distinct cell lines. The output of the X-ray irradiator was calibrated using a dosimetry-based procedure, and the dose rate was routinely verified across experimental sessions to ensure reproducibility. At 6, 12, and 24 h after irradiation, cells were fixed with 4% paraformaldehyde for 10 min at room temperature. After washing three times with wash buffer (1× PBS, 0.05% Tween-20), cells were permeabilized with 0.1% Triton X-100 in PBS for 5 min. After two additional washes, cells were blocked with 5% goat serum (Nichirei Biosciences, Tokyo, Japan) for 30 min. Cells were incubated with mouse anti-phospho-H2A.X (Ser139) IgG (1:500; Millipore, Burlington, MA, USA) [19] for 2 h at room temperature, washed three times, and then incubated with Alexa Fluor 488-conjugated goat anti-mouse IgG (1:1000; Invitrogen, Thermo Fisher Scientific, Waltham, MA, USA) for 1 h at room temperature. After three washes, slides were mounted with ProLong Diamond Antifade Mountant with DAPI (Invitrogen, Thermo Fisher Scientific, Waltham, MA, USA). Fluorescence images were acquired using a FLoid Cell Imaging Station (Thermo Fisher Scientific, Waltham, MA, USA) with DAPI (blue) and 488 (green) channels. Exposure time and gain were kept constant, and images were saved using the default device settings. Two random fields per well were captured, excluding edge regions. γH2AX signal quantification was performed using ImageJ (NIH) [16]. Nuclear regions of interest (nuclear ROIs) were extracted from DAPI images, and 488-channel signals within the ROIs were quantified automatically using a custom macro. Nuclei with 488-channel signals meeting a fixed threshold (max intensity ≥ 1800) were defined as γH2AX-positive, and the percentage of γH2AX-positive cells was calculated using DAPI-identified nuclei as the denominator. The fixed threshold (max intensity ≥ 1800) was empirically determined based on the upper limit of nuclear background signals observed in non-irradiated (0 Gy) controls, i.e., set above the maximal background intensity to minimize false positives while enabling clear separation from irradiation-induced γH2AX-positive nuclei. The same imaging conditions and threshold were applied uniformly across all groups and time points to avoid analyst-dependent bias. At least two wells were used per condition and time point, and experiments were independently repeated three times.

### 4.10. Clonogenic Survival Assay for Radiosensitivity

Radiosensitivity of LEA/LEC cells and their stable HMCES-expressing lines was evaluated by clonogenic survival after X-ray irradiation [20]. Exponentially growing cells were treated with TrypLE Express (Invitrogen, Thermo Fisher Scientific, Waltham, MA, USA) to obtain a single-cell suspension, and viability was confirmed to be ≥90% by trypan blue exclusion prior to experiments. Cells were seeded into 60-mm tissue culture dishes and cultured at 37 °C with 5% CO_2_. Seeding densities for each dose and cell line were optimized in preliminary experiments to yield 20–150 colonies per dish. Depending on the dose, seeding densities were typically set within 10^2^–10^4^ cells/dish, and the actual number of cells seeded in each experiment was recorded for calculation of the surviving fraction (SF). After seeding, dishes were left undisturbed for at least 6 h at 37 °C with 5% CO_2_ to allow for uniform attachment and resumption of proliferation and then irradiated. Irradiation was performed using the MX-80Labo system (mediXtec, Matsudo, Japan) at 80 kV and 1.25 mA, with a source-to-dish-bottom distance of 110 mm and no filter. The 0 Gy group was placed in the irradiator without exposure (sham irradiation). After irradiation, cells were cultured for 7 days at 37 °C with 5% CO_2_. Culture medium was removed, dishes were fixed with methanol for 10 min, and stained with 0.25% crystal violet (Sigma-Aldrich, St. Louis, MO, USA) for 1–2 min. After washing off excess stain with running water, dishes were completely air-dried. Colonies were defined as distinct cell clusters containing ≥50 cells and counted visually using the same criteria across all conditions. In this study, *n* = 6 was treated as independent biological replicates (repeats performed on different days with independent cell preparations and independently executed irradiation, culture, and counting procedures).

### 4.11. Calculation of Surviving Fraction (SF) and Linear–Quadratic (LQ) Model Fitting

Plating efficiency (PE) was calculated from the number of colonies and the number of cells seeded in the 0 Gy (non-irradiated) group as follows: PE (%) = {number of colonies (0 Gy)/number of cells seeded (0 Gy)} × 100 [6]. For each dose D (Gy), SF was calculated by normalizing the colony count per seeded cell to the PE of the 0 Gy group: SF(D) = {number of colonies (D)/number of cells seeded (D)}/{PE (0 Gy)/100}. SF values at each dose were fitted to the linear–quadratic (LQ) model, SF(D) = exp(−αD − βD^2^), using nonlinear least squares regression. For visualization, SF was plotted on a logarithmic scale.

### 4.12. Quantification of End-Joining Activity Using the pimEJ5GFP Reporter

To examine whether HMCES is involved in double-strand break (DSB) repair, particularly the end-joining pathway, the pimEJ5GFP reporter assay was performed [23]. The pimEJ5GFP plasmid (Addgene plasmid #44026, Watertown, MA, USA) was obtained from the depositor. In this reporter system, introduction of a DSB by I-SceI restores GFP expression upon end joining, and the proportion of GFP-positive cells is primarily interpreted as an indicator of classical non-homologous end joining (c-NHEJ) activity. However, because the readout may reflect overall end-joining activity, the contribution of alternative end-joining pathways cannot be completely excluded. The DSB substrate was prepared by digesting the pimEJ5GFP plasmid with I-SceI (New England Biolabs, Ipswich, MA, USA), and cleavage was confirmed by agarose gel electrophoresis. A total of 293 WT and 293 HMCES-KO cells were seeded in 6-well plates and co-transfected with the linearized pimEJ5GFP plasmid (2.5 µg) and CMV-RFP (Addgene plasmid #17619, Watertown, MA, USA) (0.1 µg) using TransIT-X2 (Mirus Bio, Madison, WI, USA). After 48 h, fluorescence images were acquired (FLoid Cell Imaging Station; Thermo Fisher Scientific, Waltham, MA, USA), and GFP-positive cells were quantified using ImageJ (NIH). To correct for transfection efficiency, GFP-positive counts were normalized to RFP-positive counts, and GFP^+^/RFP^+^ (%) was calculated as end-joining activity.

### 4.13. Statistical Analysis

Statistical analyses were performed primarily using EZR (Easy R; Saitama Medical Center, Jichi Medical University). For comparisons between two groups, Welch’s *t*-test (two-sided) was used without assuming equal variances. For analyses involving three or more groups or multiple conditions, one-way analysis of variance (ANOVA) was performed, and Holm’s method was applied for multiple-comparison adjustment when appropriate. For the EJ5-GFP reporter assay, one-way ANOVA was followed by planned comparisons using the WT group as the reference, with Holm’s method for adjustment. For γH2AX immunofluorescence analysis, time points were treated as independent evaluation axes, and between-group comparisons were performed at each time point. For RNA-seq, statistical significance of expression changes was computed using DESeq2 and evaluated using Benjamini–Hochberg-adjusted *p*-values (FDR). RNA-seq was positioned as an exploratory analysis for prioritization of candidate genes, and results were interpreted in light of the limited sample size. SF data obtained from the clonogenic assay were fitted to the LQ model by nonlinear least squares regression. In all analyses, *p* < 0.05 was considered statistically significant. Unless otherwise noted, data are presented as mean ± standard deviation (SD), and error bars indicate SD.

## Data Availability

The original contributions presented in this study are included in the article/Supplementary Materials. Further inquiries can be directed to the corresponding author.

## Supplementary Materials

The following supporting information can be downloaded at https://www.mdpi.com/article/10.3390/ijms27031278/s1.
